# *In vitro* study of the Polo‐like kinase 1 inhibitor volasertib in non‐small‐cell lung cancer reveals a role for the tumor suppressor p53

**DOI:** 10.1002/1878-0261.12477

**Published:** 2019-04-05

**Authors:** Jolien Van den Bossche, Christophe Deben, Ines De Pauw, Hilde Lambrechts, Christophe Hermans, Vanessa Deschoolmeester, Julie Jacobs, Pol Specenier, Patrick Pauwels, Jan Baptist Vermorken, Marc Peeters, Filip Lardon, An Wouters

**Affiliations:** ^1^ Center for Oncological Research (CORE) University of Antwerp Wilrijk Belgium; ^2^ Department of Pathology Antwerp University Hospital Edegem Belgium; ^3^ Department of Oncology Antwerp University Hospital Edegem Belgium

**Keywords:** hypoxia, non‐small‐cell lung cancer, p53, Polo‐like kinase 1, senescence, volasertib

## Abstract

Polo‐like kinase 1 (Plk1), a master regulator of mitosis and the DNA damage response, is considered to be an intriguing target in the research field of mitotic intervention. The observation that Plk1 is overexpressed in multiple human malignancies, including non‐small‐cell lung cancer (NSCLC), gave rise to the development of several small‐molecule inhibitors. Volasertib, presently the most extensively studied Plk1 inhibitor, has been validated to efficiently reduce tumor growth in preclinical settings. Unfortunately, only modest antitumor activity against solid tumors was reported in clinical trials. This discrepancy prompted research into the identification of predictive biomarkers. In this study, we investigated the therapeutic effect of volasertib monotherapy (i.e., cytotoxicity, cell cycle distribution, apoptotic cell death, cellular senescence, and migration) in a panel of NSCLC cell lines differing in p53 status under both normal and reduced oxygen tension (<0.1% O_2_). A strong growth inhibitory effect was observed in p53 wild‐type cells (A549 and A549‐NTC), with IC_50_ values significantly lower than those in p53 knockdown/mutant cells (A549‐920 and NCI‐H1975) (*P* < 0.001). While mitotic arrest was significantly greater in cells with nonfunctional p53 (*P* < 0.005), apoptotic cell death (*P* < 0.026) and cellular senescence (*P* < 0.021) were predominantly induced in p53 wild‐type cells. Overall, the therapeutic effect of volasertib was reduced under hypoxia (*P* < 0.050). Remarkably, volasertib inhibited cell migration in all cell lines tested (*P* < 0.040), with the exception of for the NCI‐H1975 p53 mutant cell line. In conclusion, our results show an important difference in the therapeutic effect of Plk1 inhibition in NSCLC cells with versus without functional p53. Overall, the p53 wild‐type cell lines were more sensitive to volasertib treatment, suggesting that p53 might be a predictive biomarker for Plk1 inhibition in NSCLC. Moreover, our results pave the way for new combination strategies with Plk1 inhibitors to enhance antitumor activity.

**Abbreviations**
ALK: anaplastic lymphoma kinase
ATCC: American Type Cell Culture Collection
EMT: epithelial‐to‐mesenchymal transition
EGFR: epidermal growth factor receptor
FBS: fetal bovine serum
FFPE: formaldehyde‐fixed paraffin‐embedded
IC_50_: half‐maximal inhibitory concentration
NSCLC: non‐small‐cell lung cancer
PARP: poly(ADP)ribose polymerase
pHH3: phospho‐histone H3
Plk1: polo‐like kinase 1
SAC: spindle assembly checkpoint
SD: standard deviation
siRNA: small‐molecule interfering RNA
SRB: sulforhodamine B
ROS: reactive oxygen species
TIS: treatment‐induced senescence
TKI: tyrosine kinase inhibitor

## 1 Introduction

In recent years, remarkable progress has been made in our understanding of molecular pathways governing tumor cell functions. This has led to an explosive interest in the development of targeted agents based on the molecular profile of individual tumors. As a result, several novel therapies such as epidermal growth receptor (EGFR) tyrosine kinase inhibitors (TKIs) (e.g., erlotinib, gefitinib, and afatinib) and anaplastic lymphoma kinase (ALK) inhibitors (e.g., crizotinib, ceritinib, and alectinib) are now at the forefront of personalized medicine for the treatment of non‐small‐cell lung cancer (NSCLC). Nevertheless, treatment options with TKIs and ALK inhibitors are limited to a minority of patients, harboring targetable EGFR or ALK mutations in their tumor cells, and rates of acquired therapy resistance remain very high in clinical settings (Rolfo *et al*., 2014). Over the last decades, chemotherapy consisting of platinum‐based doublets remains the first‐line treatment for the majority of advanced NSCLC patients (Chen *et al*., 2014). However, as the standard combination chemotherapies have reached a plateau in treatment efficacy with median overall survival rates of approximately 8 months and overall response rates of 19% to 30%, most patients without targetable mutations are now treated with immunotherapeutic agents, including immune checkpoint inhibitors such as antiprogrammed cell death 1 (PD‐1) antibodies (e.g., nivolumab and pembrolizumab) (Pallis *et al*., 2009) (Zhang *et al*., 2019).

Agents that affect the mitotic spindle (e.g., taxanes and vinca alkaloids) are well‐established components of these standard combination chemotherapy treatment schedules. Though, serious adverse effects caused by interactions with the tubulin cytoskeleton in nondividing differentiated cells remain the dose‐limiting factor (Jackson *et al*., 2007; Strebhardt, 2010). Advances in our knowledge of mitotic cell division have resulted in the identification of several crucial regulatory proteins, leading to the development of mitosis‐specific therapeutics. An interesting target in this research field is Polo‐like kinase 1 (Plk1), the founding member of the Plk family (Plk1‐5) (Christoph and Schuler, 2011). Plk1 regulates multiple steps during mitosis, from mitotic entry to centrosome maturation, bipolar spindle formation, activation of the anaphase‐promoting complex, chromosome segregation, and initiation of cytokinesis (Guan *et al*., 2005; Louwen and Yuan, 2013; Van den Bossche *et al*., 2016). Consistent with its mitotic functions, the expression and activity of Plk1 are low during the G_0_/G_1_ and S phase, begin to rise through G_2_ phase, and peak in M phase of the cell cycle. Balanced Plk1 expression levels are crucial to ensure proper cell division. Upregulation of Plk1 expression and/or activity might lead to the formation of aberrant centrosomes, incorrect mitotic spindles, or defective cell cycle checkpoints, resulting in chromosomal instability and aneuploidy (Sanhaji *et al*., 2012). The observed overexpression of Plk1 in several human malignancies, including non‐small‐cell lung cancer (NSCLC), and its correlation with poor patient prognosis has led to the development of various small‐molecule inhibitors against Plk1 (Li *et al*., 2017; Strebhardt and Ullrich, 2006; Van den Bossche *et al*., 2017; Wang *et al*., 2012). Plk1 inhibition by volasertib, presently the most extensively studied Plk1 inhibitor, reduced tumor growth with a high efficacy in both *in vitro* and *in vivo* settings. Nevertheless, modest antitumor activity in solid tumors was observed in clinical studies. The discrepancy between the preclinical data and clinical outcome prompted the research into the identification of predictive biomarkers for Plk1 inhibition. In this regard, the tumor suppressor p53, which ensures regulation of the response to cellular stress signals by induction of cell cycle arrest, apoptosis, or senescence, has previously been described as a potential candidate (Sanhaji *et al*., 2012, 2013). In addition to activating the expression of a wide array of genes involved in tumor‐protecting processes, p53 also represses the expression of many genes required for cell cycle progression and survival (McKenzie *et al*., 2010). As such, the Plk1 promotor is downregulated by p53 upon DNA damage, leading to mitotic arrest and the induction of apoptotic cell death. On the other hand, Plk1 also negatively regulates p53, thereby inhibiting its proapoptotic function (Liu *et al*., 2006; Louwen and Yuan, 2013). As a result, the functionality of p53 might be crucial for the susceptibility of cancer cells to Plk1 inhibition. Previously, we identified Plk1 expression combined with the *TP53* mutation status and the occurrence of hypoxic regions as a promising prognostic biomarker panel for NSCLC (Van den Bossche *et al*., 2017). However, no conclusive evidence is found in literature about their predictive role for Plk1 inhibition.

Here, we evaluated the therapeutic response of volasertib in the NSCLC cell lines A549 (p53 wild‐type) and its isogenic derivatives A549‐NTC (nontemplate control, p53 wild‐type) and A549‐920 (p53 shRNA knockdown), as well as in the TP53 mutant NCI‐H1975 cell line (R273H). We investigated the effect of Plk1 inhibition on cell growth, cell cycle distribution, induction of cell death, cellular senescence, and the migratory potential. Experiments were performed under both normal and reduced oxygen conditions (i.e., hypoxia) as hypoxic tumor regions are likely to be more resistant to therapy (Wouters *et al*., 2009) and no information about the cytotoxic potential of volasertib under hypoxia is available yet.

## 2 Materials and methods

### 2.1 Cell lines and cell culture

The NSCLC adenocarcinoma cell lines A549 (CCL‐185, *TP53* ^WT^) and NCI‐H1975 (CRL‐5908, mutant nonfunctional p53, *TP53* ^R273H^) were obtained from the American Type Cell Culture Collection (ATCC, Rockville MD, USA) and cultured in DMEM and RPMI medium, respectively, supplemented with 10% fetal bovine serum (FBS), 1% penicillin/streptomycin, and 1% l‐glutamine. Additionally, 1% sodium pyruvate was added to the RPMI medium. All cell culture reagents were purchased from Life Technologies (Ghent, Belgium). Cells were maintained as monolayers in exponential growth in a 5% CO_2_/95% O_2_ humidified incubator at 37 °C and confirmed free of mycoplasma contamination through regular testing (MycoAlert^®^ Mycoplasma Detection Kit, Lonza, Verviers, Belgium). In order to verify the role of p53 in response to Plk1 inhibition, the parental A549 cell line was transduced with a GIPZ lentiviral shRNA VGH5526‐EG7157 viral particle set (Thermo Scientific, Erembodegem, Belgium), as described previously (Deben *et al*., 2015). The isogenic derivatives A549‐NTC (nontemplate control, functional p53) and A549‐920 (p53 shRNA, p53 knockdown) were continuously exposed to puromycin (1 μL·mL^−1^) (InvivoGen, Toulouse, France) to obtain stably transduced cell lines. P53 expression levels were tested on a regular base using western blot (Fig. S1). For subsequent experiments, cells were harvested by trypsinization, automatically counted with a Scepter 2.0 device (Merck Millipore, Overijse, Belgium), and plated as specified below.

### 2.2 Oxygen conditions

Hypoxic conditions (0% O_2_, 5% CO_2_, and 95% N_2_) were achieved in a Bactron IV anaerobic chamber (Shel Lab, Cornelius, USA). Measurements with a ToxiRAE II air oximeter (RAE BeNeLux, Hoogstraten, Belgium) confirmed that the oxygen tension in the gas phase was stable at < 0.1% O_2_. Cells were incubated under hypoxia for 24 or 72 h immediately after treatment.

### 2.3 Sulforhodamine b assay: cytotoxicity

Cells were plated in 96‐well plates, and after an overnight recovery period under normoxia, cells were treated with 0 ‐ 85 nm volasertib (Selleck Chemicals, Huissen, The Netherlands) for 24 or 72 h and incubated either under normal or reduced oxygen tension. Seventy‐two hours after the start of the treatment, cell survival was determined using the SRB assay, as previously described (Pauwels *et al*., 2003). The half‐maximal inhibitory concentration (IC_50_), representing the drug concentration leading to 50% growth inhibition, was calculated using winnonlin Software (Pharsight, Mountain View, USA).

### 2.4 Western blot: plk1, p21, and p53

Cells were lysed on 6‐well plates in TNN buffer 72 h after treatment with volasertib (0–20 nm). After centrifugation (10 min, 17 949 *g*, 4 °C), the supernatant containing the isolated proteins was stored at −80 °C. Protein concentrations were determined using the Pierce^®^ BCA protein kit (Thermo Scientific). Twenty microgram of proteins was separated by SDS/PAGE, and western blot was performed as described previously (Deben *et al*., 2016). Blocking, primary and secondary antibody incubation were performed using the SNAP i.d. ^®^ 2.0 Protein Detection System (Merck Millipore) according to the manufacturer's instructions. Membranes were incubated with the following antibodies: rabbit monoclonal anti‐Plk1 (1 : 500, Cell Signaling, , Leiden, The Netherlands), rabbit monoclonal anti‐p53 (1 : 2000, Cell Signaling, Leiden, The Netherlands, no. 9282), and rabbit monoclonal anti‐p21 (1 : 2000, Abcam, Cambridge, UK, no. 109199). Anti‐β‐actin was used as internal standard (1 : 5000, Sigma‐Aldrich, Diegem, Belgium no. A5441). Goat anti‐rabbit (1 : 1000, Li‐Cor, Leusden, The Netherlands, no. 926‐32211) or goat anti‐mouse (1 : 10000, Li‐Cor, no. 926‐68070) fluorescently labeled secondary antibody was used (1 : 10000), and fluorescent detection was performed using an Odyssey imaging system (Li‐Cor). Protein levels were quantified using image studio™ lite, a software program available on the Odyssey imaging system. Plk1, p53, and p21 expression levels were corrected for loading differences based on β‐actin expression. Three independent protein isolations were performed in order to ensure reproducibility of the results.

### 2.5 Vindelov method: cell cycle distribution

Cells were seeded in 6‐well plates, and after an overnight recovery period, cells were treated for 24 h with vehicle, 7.5 nm, 12.5 nm, or 20 nm volasertib, corresponding with the IC_20_, IC_40_, and IC_60_ values in A549 cells, respectively. Cell cycle analysis was performed immediately after the treatment period using the CycleTEST™ PLUS DNA Reagent Kit (Becton Dickinson, Erembodegem, Belgium), according to the manufacturer's instructions. Flow cytometric analysis was performed on a FACScan flow cytometer (Becton Dickinson). Each sample was analyzed using 10.000 events/sample acquired. Histograms of DNA content were analyzed using flowjo Software v.10.0.7 to determine the percentage of cells in each phase of the cell cycle.

### 2.6 Annexin v/pi staining: apoptotic cell death

Cells were plated in 6‐well plates, incubated overnight, and treated for 72 h with vehicle, 7.5 nm, 12.5 nm, or 20 nm volasertib. Seventy‐two hours later, apoptotic cell death was evaluated using the Annexin V‐FITC Apoptosis Detection Kit (Becton Dickinson), according to the manufacturer's instructions. Flow cytometric analysis was performed on a FACScan flow cytometer (Becton Dickinson). Each sample was analyzed using 10.000 events/sample acquired. Data were analyzed using flowjo Software v.10.0.7. For the A549‐NTC and A549‐920 cell lines, the number of apoptotic cells was determined using Annexin V‐PerCP due to the interference of FITC with the control protein turbo‐GFP, present in the vector.

### 2.7 Cleaved caspase 3/7 staining: apoptotic cell death

Alternatively, the induction of apoptotic cell death was monitored in real time using the IncuCyte^®^ ZOOM live‐cell analysis instrument, equipped with a 10x objective (Essen Bioscience, MI, USA). Cells were plated in 96‐well plates, and after an overnight recovery period, cells were treated with volasertib (0–50 nm) for 72 h. The IncuCyte^®^ Caspase 3/7 Green Apoptosis Reagent (Essen Bioscience, Cat no. 4440) was added at the start of the treatment at a final concentration of 2.5 μm. Images were taken every 2 h after treatment start. Analysis was performed using the IncuCyte^®^ ZOOM 2016B software. Data are presented as green object counts·mm^−2^.

### 2.8 β‐Galactosidase staining: cellular senescence

Cells were seeded in 6‐well plates before treatment with volasertib (0–20 nm) for 24 h. Seventy‐two hours later, cells were fixed and stained at pH 6.0 using a senescence β‐galactosidase kit (Cell Signaling, no. 9860). Plates were incubated with X‐gal staining solution overnight at 37 °C in a dry incubator without CO_2_. Using a transmitted‐light microscope (Olympus BX41), equipped with a Leica DFC450C camera, blue staining was visualized in three random nonoverlapping fields using a 10x objective with a 10× eyepiece for quantification. The percentage of senescent cells (morphological changes combined with blue staining) was determined using imagej software.

### 2.9 Immunofluorescence: phospho‐histone h3 (phh3) and phospho‐histone h2ax (γ‐h2ax) staining

In order to confirm the induction of mitotic arrest and/or DNA damage after Plk1 inhibition, immunofluorescence experiments using the mouse monoclonal anti‐pHH3 (Ser10) antibody (1 : 2000, Merck Millipore, no. 05‐806) and anti‐γ‐H2AX (Ser139) antibody (1 : 500, Merck Millipore, no. 05‐636‐AF488) were performed. Seventy‐two hours (γ‐H2AX) or 24 h (pHH3) after treatment with volasertib (0–50 nm), cells were fixed with ice‐cold methanol, permeabilized with 0.1% Triton X‐100/PBS, and blocked with 1% BSA/PBS for 1 h. Next, cells were incubated overnight with the primary antibody at 4 °C, followed by 1‐h incubation at room temperature with the secondary antibody, that is, donkey anti‐mouse IgG Alexa Fluor^®^ 555 conjugate (1 : 1000, Thermo Scientific). Slides were counterstained with DAPI and mounted. Images of sections stained with the anti‐pHH3 antibody were taken using an Olympus BX51 standard research fluorescence microscope (Olympus, Aartselaar, Belgium), equipped with an Olympus DP71 digital camera (Olympus). Sections stained with the anti‐γ‐H2AX antibody were visualized with an Evos Cell Imaging System (Thermo Scientific). The percentage of positive pHH3 cells and the amount of γ‐H2AX foci per cell were counted using imagej software.

### 2.10 Transwell migration experiment

Cell migration experiments were performed using a conventional 24‐well Transwell system (10 μm thickness, 8 μm pores) (Corning^®^, New York, USA). In brief, after 24 h pretreatment with vehicle or volasertib (0–20 nm), cells were detached using TrypLE™ Express (Invitrogen, Erembodegem, Belgium), counted, and resuspended in serum‐free medium. A volume of 250 μL containing 50.000 cells was seeded to each insert, and 600 μL medium supplemented with 10% FBS was added to the lower wells. Eight hours later, cells were fixed and stained, as described previously (Limame *et al*., 2012). Stained membranes were visualized in three random nonoverlapping fields at 10x objective and 10x eyepiece on an Olympus BX41 transmitted‐light microscope (Olympus), equipped with a Leica DFC450C camera (Leica Microsystems, Diegem, Belgium). Quantification was performed by processing all obtained images using imagej software. Degree of migration per well was determined by calculating the average pixel area of the three fields in triplicate.

### 2.11 Statistical analysis

All experiments were performed at least three times. Results are presented as mean ± standard deviation (SD). All statistical analyses were conducted using spss v23 and v25 (SPSS Inc., Brussels, Belgium); whereby, a *P*‐value < 0.05 was considered as statistically significant. Statistical significances of the migration experiments were determined by a one‐way ANOVA test. An unpaired Student's *t*‐test or two‐way ANOVA test was used for all other experiments to evaluate the impact of cell line, volasertib concentration, treatment duration, and oxygen tension on the outcome parameter (survival, cell cycle distribution, mitotic index, cell death, senescence, and DNA damage). A Tukey post hoc comparison test revealed which groups differed significantly from one other.

## 3 Results

### 3.1 The responsiveness to volasertib is significantly influenced by the tp53 status

**Figure 1:**
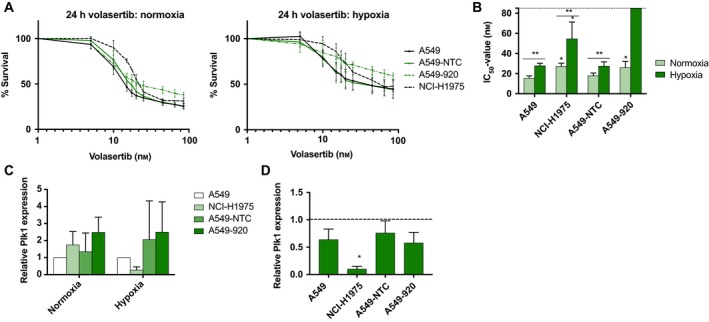
Growth inhibition upon volasertib treatment is p53‐dependent. (A) Dose–response curves after 24‐h treatment with volasertib (0–85 nm) under normoxic and hypoxic conditions in the p53 wild‐type cell lines A549 and A549‐NTC, the p53 knockdown cell line A549‐920, and the *TP*53 mutant cell line NCI‐H1975. Data are presented as mean ± SD of at least three independent experiments. (B) IC_50_ values after 24‐h treatment with volasertib. *P‐value < 0.05 compared to A549 for NCI‐H1975 cells and to A549‐NTC for A549‐920 cells (unpaired Student's *t*‐test). ** *P*‐value < 0.05 between normoxia and hypoxia (unpaired Student's *t*‐test). IC_50_ values in all tested conditions are presented in Table**** 1. (C) Baseline Plk1 expression in *TP53* wild‐type and deficient/mutant cell lines under both normal and reduced oxygen conditions. Results are presented as mean ± standard deviation of at three independent experiments. Plk1 expression levels are normalized to the A549 cell line. (D) Baseline Plk1 expression in *TP53* wild‐type and deficient/mutant cell lines under hypoxic condition. Results are presented as mean ± standard deviation of at three independent experiments. For each cell line, Plk1 expression is normalized to the Plk1 levels in untreated samples under normoxia. *P‐value < 0.05 compared to normoxia (unpaired Student's *t*‐test).

To evaluate the role of functional p53 on the effect of Plk1 inhibition, NSCLC cells with different p53 status were treated with volasertib (0–85 nm) for 24 or 72 h, whereafter survival was assessed using the SRB assay. A clear concentration‐dependent growth inhibition effect of volasertib was observed in all NSCLC cell lines, under both normal and reduced oxygen conditions (Fig. 1A). In general, the p53 wild‐type cell line A549 and its nontemplate control A549‐NTC were more sensitive to the Plk1 inhibitor, with IC_50_ values significantly lower than the isogenic p53 knockdown cell line A549‐920 and the *TP53* mutant cell line NCI‐H1975 (*P* < 0.001). No significant difference was observed between the A549 parental cell line and the A549‐NTC control cell line (*P* = 0.795). Statistical analysis showed no significant difference between 24 h and 72 h of treatment, suggesting that the duration of treatment did not affect the cytotoxic effect of volasertib (*P* > 0.172). Fig. 1B and Table 1 represent the IC_50_ values under both normoxia and hypoxia for all cell lines.

**Table 1:** IC_50_ values after Plk1 inhibition in the p53 wild‐type cell lines A549 and A549‐NTC, the p53 knockdown cell line A549‐920, and the p53 mutant cell line NCI‐H1975. Cells were treated with a concentration range of volasertib (0–85 nm) for 24 h or 72 h and incubated under both normal and reduced oxygen conditions. Cell survival was determined using the SRB assay, and IC_50_ values were calculated using winnonlin software. All data are presented as mean ± standard deviation of at least three independent experiments

	IC_50_ values 24‐h volasertib treatment (nm)			
	A549	A549‐NTC	A549‐920	NCI‐1975
Normoxia	15.54 ± 2.17	18.05 ± 2.52	26.14 ± 5.93	27.29 ± 3.09
Hypoxia	27.85 ± 2.42	27.56 ± 4.16	NR	54.71 ± 16.61
	IC_50_ values 72‐h treatment volasertib treatment (nm)			
	A549	A549‐NTC	A549‐920	NCI‐1975
Normoxia	17.79 ± 2.15	17.57 ± 1.27	24.88 ± 1.96	21.30 ± 2.54
Hypoxia	21.7 ± 1.97	30.06 ± 1.95	NR	53.28 ± 11.91

IC_50_, half‐maximal inhibitory concentration; NR, not reached.

Overall, calculation of the IC_50_ value revealed that oxygen deficiency negatively influenced the effectiveness of the Plk1 inhibitor. After 24 h of treatment, IC_50_ values were significantly higher in hypoxic cells compared to their normoxic counterparts (*P* < 0.05). For example, in A549‐NTC cells, the IC_50_ value was 18.05 ± 2.52 nm under normoxia and 27.56 ± 4.16 nm when cells were incubated in the hypoxic chamber. In the A549‐920 cell line, volasertib treatment with concentrations up to 85 nm resulted in 58.88 ± 3.28% cell survival under hypoxia, while only 37.95 ± 5.04% of the cells survived under normal oxygen tension. As the IC_50_ value was not reached in this cell line under hypoxic conditions, no statistical analysis could be done for this condition. As such, the effect of the p53 status on cell survival after Plk1 inhibition was further enhanced under hypoxia. NSCLC cells with nonfunctional p53 were significantly less sensitive to Plk1 inhibition under hypoxia compared to their normoxic counterparts (*P* < 0.004).

In order to evaluate whether the difference in sensitivity to volasertib was due to a difference in baseline Plk1 protein expression, western blot using an anti‐Plk1 antibody was performed on untreated samples (Fig. 1C). No significant differences in Plk1 expression could be observed between the cell lines, neither under normoxia (*P* > 0.182) nor under hypoxia (*P* > 0.301). Interestingly, a decrease in Plk1 expression was noticed when cells were cultured under reduced oxygen conditions (Fig. 1D), but this effect was only significant in NCI‐H1975 cells (p_A549 _= 0.079, p_NCI‐H1975 _= 0.001, p_A549‐NTC _= 0.210, and p_A549‐920 _= 0.059).

Moreover, in order to investigate the molecular mechanisms behind the observed differences in sensitivity to volasertib treatment under normal versus reduced oxygen tensions, we determined the protein expression of hypoxia‐inducible factor 1α (HIF‐1α), the major player in the response to hypoxia. HIF‐1α acts as a transcription factor for genes involved in cell proliferation, cell cycle, apoptosis, cellular metabolism, and angiogenesis, thus promoting cell survival under hypoxia. As expected, HIF‐1α expression levels were significantly upregulated when untreated cells were incubated under reduced oxygen tension (*P* < 0.024). Treatment with volasertib did not induce a significant increase in HIF‐1α expression, in all cell lines tested, neither under normoxia nor under hypoxia (*P* > 0.096). Nevertheless, a strong trend for higher HIF‐1α expression levels was observed when comparing p53 nonfunctional/mutant cells versus p53 wild‐type cells after treatment with volasertib under hypoxic conditions (*P* = 0.057).

### 3.2 Volasertib treatment results in mitotic arrest

**Figure 2:**
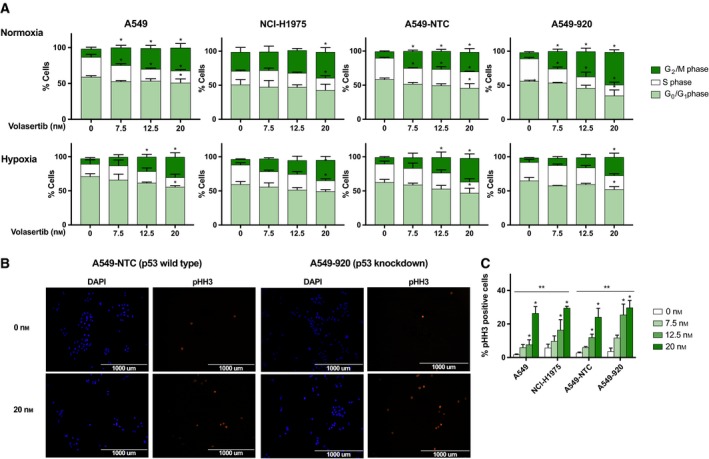
G_2_/M cell cycle arrest after treatment with volasertib is more pronounced in NSCLC cell lines without functional p53. (A) Cells were treated with volasertib (0–20 nm) under normoxia and hypoxia and stained with PI 24 h after the start of the treatment. DNA content was determined by flowcytometric analysis. Cells were divided into three groups: G_1_ phase (2N), S phase (2N‐4N), and G_2_/M phase (4N). The percentage of cells in each phase is presented as mean ± SD of at least three independent experiments. *P‐value < 0.05 compared to untreated sample (one‐way ANOVA). (B) Immunofluorescent staining of the mitotic marker pHH3 (red) 24 h after treatment with volasertib (0–20 nm) in A549‐NTC (p53 wild‐type) and A549‐920 (p53 knockdown) cells. Nuclei were stained with DAPI in blue (40x). Scale bars are shown on the pictures (1000 μm). (C) The percentage of pHH3‐positive cells immediately after 24‐h treatment with the Plk1 inhibitor (0–20 nm) in both p53 functional (A549, A549‐NTC) and p53 nonfunctional (NCI‐H1975, A549‐920) cells. *P‐value < 0.05 compared to untreated sample (one‐way ANOVA). **P‐value < 0.05 compared to A549 for NCI‐H1975 cells and to A549‐NTC for A549‐920 cells (two‐way ANOVA).

As Plk1 is a major regulator of mitotic cell division, the cell cycle distribution was investigated immediately after treatment with volasertib (0–20 nm, 24 h). As presented in Fig. 2A, exposure to the Plk1 inhibitor caused a significant G_2_/M phase block in all NSCLC cell lines (*P* < 0.047), accompanied by a significant decrease in number of G_1_ and S phase cells (both *P* < 0.001). The G_2_/M arrest was clearly influenced by the volasertib concentration (*P* < 0.050), the p53 status of the cell line (*P* < 0.001), and the oxygen tension (*P* < 0.001) (Table 2). Post hoc analysis revealed that the increase in % G_2_/M phase cells induced by higher concentrations of volasertib (12.5–20 nm) was less pronounced in p53 wild‐type NSCLC cells compared to cells without functional p53, at least under normoxia. For example, whereas the percentage of G_2_/M cells increased from 9.09 ± 1.11% (0 nm) to 47.77 ± 3.53% (20 nm) in the A549‐920 cell line, there was a smaller increase from 9.66 ± 0.84% to 28.53 ± 5.25% in A549‐NTC cells (*P* < 0.037) under normoxia. No significant difference was observed between A549 cells and A549‐NTC cells (*P* > 0.674). When cells were exposed to hypoxia, an accumulation in the G_2_/M phase was only detected when high volasertib concentrations (12.5 nm or 20 nm) were used. Post hoc analysis revealed no significant differences in mitotic arrest after volasertib treatment between p53 wild‐type and p53‐deficient/mutant cell lines under hypoxia (*P* > 1.09). In untreated control cells, oxygen deficiency resulted in a significant increase in G_1_ phase cells (*P* < 0.001).

**Table 2:** Results of post hoc analysis of the percentage of cells in the G_2_/M phase of the cell cycle depending on cell line, drug concentration, and oxygen tension. Cells were treated with a concentration range of volasertib (0–20 nm) for 24 h under both normal and reduced oxygen conditions. Percentages of G_2_/M phase cells were determined using flow cytometry. Upper row: *P*‐values of untreated and treated samples compared between p53 wild‐type (A549, A549‐NTC) and p53‐deficient/knockdown (A549‐920, NCI‐H1975) cells. Middle row: *P*‐values of untreated samples versus treated samples in a cell line. Lower row: *P*‐values of untreated and treated samples under normal versus reduced oxygen tension in a cell line

	*P*‐values between cell lines							
	Normoxia				Hypoxia			
	0 nm	7.5 nm	12.5 nm	20 nm	0 nm	7.5 nm	12.5 nm	20 nm
A549 vs. NCI‐H1975	*0.006*	0.585	0.823	*0.040*	0.932	0.109	0.843	0.984
A549‐NTC vs. A549‐920	0.518	0.728	*0.031*	*0.006*	*0.006*	0.247	0.175	0.192
	*P*‐values untreated versus treated							
	Normoxia			Hypoxia				
	7.5 nm	12.5 nm	20 nm	7.5 nm	12.5 nm	20 nm		
A549	*0.004*	*<0.001*	*<0.001*	0.578	*0.020*	*<0.001*		
NCI‐H1975	0.827	0.513	*0.050*	0.194	0.135	*0.021*		
A549‐NTC	*0.001*	*0.001*	*<0.001*	0.596	*0.012*	*0.001*		
A549‐920	*0.002*	*<0.001*	*<0.001*	0.501	0.120	*0.001*		
	*P*‐values normoxia versus hypoxia							
	0 nm	7.5 nm	12.5 nm	20 nm				
A549	0.094	*0.005*	0.054	0.652				
NCI‐H1975	*0.032*	0.265	0.190	0.724				
A549‐NTC	*0.003*	*0.001*	*0.018*	*0.034*				
A549‐920	0.565	*0.056*	*0.041*	*0.037*				

Italic values indicate significance.

Immunofluorescence experiments confirmed a mitotic arrest after volasertib treatment (0–20 nm), as seen by positive staining for pHH3 (Ser10) (Fig. 2B,C). In accordance with the flow cytometry data, the mitotic index was significantly higher in cells with nonfunctional p53 cells compared to their normal counterparts (*P* < 0.005). For example, after treatment with 20 nm volasertib, 29.79 ± 4.24% of the A549‐920 cells stained positive for pHH3, versus 24.13 ± 5.22% in the A549‐NTC cell line.

**Figure 3:**
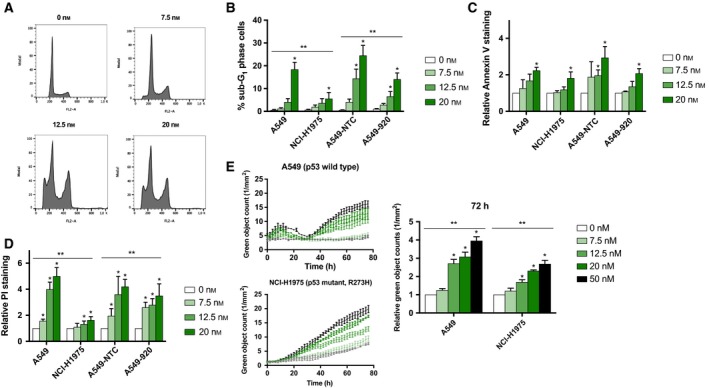
Induction of cell death is more prominent in NSCLC cell lines with functional p53 after volasertib monotherapy. (A) Cell cycle histograms of A549 cells treated with volasertib (0–20 nm) for 24 h. (B) Percentage of A549 (p53 wild‐type), NCI‐H1975 (p53 mutant), A549‐NTC (p53 wild‐type), and A549‐920 (p53 knockdown) cells in the sub‐G_1_ phase of the cell cycle after 24‐h Plk1 inhibition (0–20 nm). (C and D) Both p53 functional and nonfunctional cells were treated with volasertib (0–20 nm) during 72 h and incubated under normal and reduced oxygen conditions. Apoptotic cell death was measured using Annexin V and propidium iodide (PI) staining and is presented as relative staining compared to untreated cells in (C) and (D), respectively. Absolute percentages of Annexin V‐ and PI‐positive cells after treatment in all tested conditions are listed in Tables 3 and 4, respectively. (E) The induction of apoptotic cell death was detected by real‐time measurements of active caspase 3/7 during volasertib treatment (0–50 nm) using the IncuCyte^®^ ZOOM system. *P‐value < 0.05 compared to untreated sample (one‐way ANOVA). **P‐value < 0.05 compared to A549 for NCI‐H1975 cells and to A549‐NTC for A549‐920 cells (two‐way ANOVA).

Previously, it was reported that Plk1 inhibition can cause mitotic slippage at high concentrations, leading to multinucleated cells (Van den Bossche *et al*., 2016). However, in the present study, volasertib treatment resulted only in a negligible increase in the > 4N population in all cell lines tested. In the A549 cell line, the DNA flow cytometric profile indicated that 0.47 ± 0.14% versus 2.97 ± 1.26% of the cell population consisted of multinucleated cells in untreated versus treated (20 nm) cells. Remarkably, the flow cytometric DNA profile did reveal a sub‐G_1_ peak after treatment with high volasertib concentrations, indicating the induction of apoptotic cell death (Fig. 3A). This percentage of sub‐G_1_ cells was significantly higher in p53 wild‐type cells compared to p53 knockdown/mutant cells (*P* < 0.004) (Fig. 3B).

### 3.3 Induction of apoptotic cell death is more pronounced in p53 wild‐type cells after plk1 inhibition

To confirm the induction of apoptotic cell death, the Annexin V/PI assay was performed immediately after 72‐h treatment with volasertib (0–20 nm). In all cell lines tested, Plk1 inhibition resulted in a dose‐dependent increase in Annexin V‐positive cells compared to untreated control cells, under both normoxic and hypoxic conditions. However, this increase in Annexin V positivity was only significant when cells were treated with the highest dose of volasertib (20 nm) (Fig. 3C, Table 3). No significant difference in the increase in Annexin V‐positive cells was observed between A549, A549‐NTC, A549‐920, and NCI‐H1975 cells (*P* > 0.337). Annexin V positivity was not influenced by the absence of oxygen (*P* > 0.101), except for the NCI‐H1975 cell line (*P* = 0.019). In the latter cell line, the increase in Annexin V positivity was significantly higher when cells were treated under normoxia compared to the cells that were treated under hypoxia. In accordance with the Annexin V staining data, volasertib led to a dose‐dependent increase in the percentage of PI‐positive cells (Fig. 3D, Table 4). Overall, a low dose of volasertib (7.5 nm) induced a significant increase in PI‐positive cells in three out of four tested cell lines, while in NCI‐1975 cells higher concentrations of volasertib (12.5–20 nm) were needed to induce the same level of PI positivity. Similar to the sub‐G_1_ population, the increase in the percentage of PI‐positive cells was significantly higher in p53 wild‐type cells compared to cells lines with nonfunctional p53 (*P* < 0.026). Induction of PI‐positive cells was not influenced by the absence of oxygen (*P* > 0.182).

**Table 3:** Percentages of Annexin V‐positive cells after treatment with volasertib in both p53 wild‐type (A549 and A549‐NTC) and p53 nonfunctional (A549‐920 and NCI‐H1975) NSCLC cell lines. Cells were treated with volasertib (0–20 nm) during 72 h and incubated under both normal and reduced oxygen conditions. Annexin V‐positive cells were evaluated using flow cytometry. Data are presented as mean ± standard deviation of at least three independent experiments

	% Annexin V‐positive cells normoxia			
	0 nm	7.5 nm	12.5 nm	20 nm
A549	5.26 ± 2.21	6.14 ± 1.50	8.65 ± 3.07	12.06 ± 6.07
A549‐NTC	4.07 ± 1.15	7.04 ± 1.65	8.17 ± 3.06	11.43 ± 0.82
A549‐920	5.81 ± 0.00	6.32 ± 0.15	7.92 ± 1.63	12.10 ± 1.49
NCI‐1975	11.77 ± 1.90	12.15 ± 1.51	13.77 ± 0.91	21.30 ± 4.99
	% Annexin V‐positive cells hypoxia			
	0 nm	7.5 nm	12.5 nm	20 nm
A549	2.91 ± 0.96	3.30 ± 1.37	6.36 ± 1.88	11.77 ± 0.33
A549‐NTC	1.60 ± 0.37	1.55 ± 0.39	3.38 ± 1.18	8.17 ± 0.51
A549‐920	3.42 ± 0.53	3.24 ± 0.88	8.60 ± 1.85	10.89 ± 2.64
NCI‐1975	1.66 ± 0.23	2.24 ± 1.24	4.06 ± 0.28	7.32 ± 0.95

**Table 4:** Percentages of propidium iodide (PI)‐positive cells after treatment with volasertib in both p53 wild‐type (A549 and A549‐NTC) and p53 nonfunctional (A549‐920 and NCI‐H1975) cells. Cells were treated with volasertib (0–20 nm) during 72 h and incubated under both normal and reduced oxygen conditions. PI‐positive cells were evaluated using flow cytometry. Data are presented as mean ± standard deviation of at least three independent experiments

	% PI‐positive cells normoxia			
	0 nm	7.5 nm	12.5 nm	20 nm
A549	4.97 ± 0.50	7.80 ± 1.41	19.17 ± 1.04	24.97 ± 0.81
A549‐NTC	6.10 ± 2.79	11.95 ± 21.08	21.08 ± 2.78	25.30 ± 2.19
A549‐920	4.90 ± 0.46	12.80 ± 1.92	14.10 ± 2.18	17.47 ± 1.42
NCI‐1975	14.00 ± 2.10	15.47 ± 1.81	18.50 ± 1.15	22.80 ± 0.66
	% PI‐positive cells hypoxia			
	0 nm	7.5 nm	12.5 nm	20 nm
A549	5.97 ± 2.30	11.20 ± 1.41	16.97 ± 4.79	35.87 ± 7.66
A549‐NTC	4.60 ± 0.98	6.82 ± 3.58	15.38 ± 7.47	25.68 ± 2.21
A549‐920	5.30 ± 1.59	9.00 ± 2.98	11.00 ± 2.86	13.70 ± 2.20
NCI‐1975	10.20 ± 1.57	12.17 ± 0.67	17.40 ± 1.21	28.53 ± 1.16

Next, we monitored caspase 3/7 activity in real time for 72 h using the IncuCyte^®^ ZOOM system, directly after treatment with volasertib (0–50 nm). As shown in Fig. 3E, a time‐ and dose‐dependent increase in caspase 3/7 activity was observed in both A549 and NCI‐H1975 cells. However, the increase in green object counts was statistically higher in the p53 wild‐type cell line compared to the p53 mutant cell line (*P* < 0.001).

### 3.4 Volasertib treatment results in cellular senescence in a partially p53‐dependent pathway

**Figure 4:**
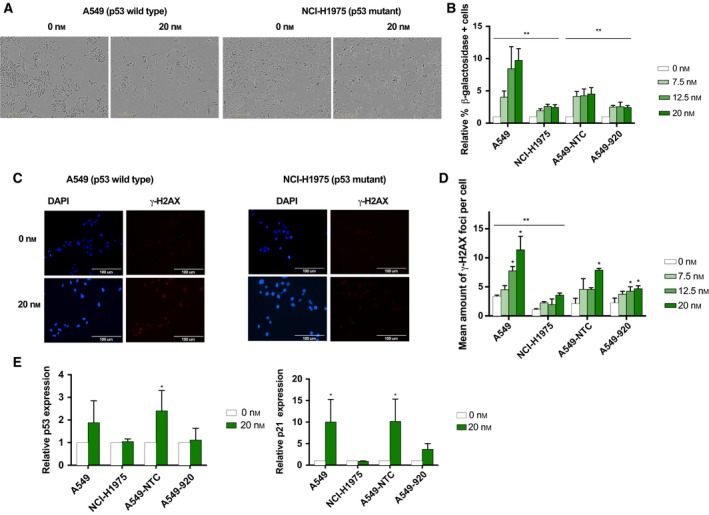
Volasertib induces premature senescence through, at least partially, a p53‐dependent pathway. (A) Changes in cell morphology (i.e., enlarged and flattened cellular size and increased vacuolization) after 24 h of treatment with volasertib (0–20 nm), followed by an incubation period for 72 h in drug‐free medium. Scale bars are shown on the pictures (300 μm). (B) Percentage of β‐galactosidase‐positive cells after Plk1 inhibition relative to untreated cells. *P‐value < 0.05 compared to A549 for NCI‐H1975 cells and to A549‐NTC for A549‐920 cells (2‐way ANOVA). (C) Immunofluorescent staining of the DNA damage marker γ‐H2AX directly after 72‐h treatment with volasertib (0–20 nm). Nuclei were stained with DAPI in blue (400x). Scale bars are shown on the pictures (100 μm). (D) Mean amount of γ‐H2AX foci per cell after 72 h of treatment with volasertib (0–20 nm) in both p53 functional and nonfunctional cells. *P‐value < 0.05 compared to untreated sample (one‐way ANOVA). **P‐value < 0.05 compared to A549 for NCI‐H1975 cells (2‐way ANOVA). (E) P53 and p21 levels were quantified after 72 h of Plk1 inhibition (0–20 nm) using western blot. β‐actin was used as an internal control. Results are corrected for loading differences based on β‐actin expression and are presented as mean ± standard deviation of at least three independent experiments. * *P*‐value < 0.05 compared to untreated sample within each cell line (unpaired Student's *t*‐test).

In addition to cell cycle arrest and apoptosis, cellular senescence has been reported to be a common outcome after treatment with antimitotic agents. Senescence, an irreversible growth arrest that occurs as a result of cellular stress or DNA damage, is characterized by morphological changes, including enlarged and flattened cellular size, increased vacuolization, expression of senescence‐associated β‐galactosidase, and nuclear foci containing DNA damage response proteins. Here, we investigated whether volasertib is able to induce cellular senescence. Both p53 wild‐type and p53 knockdown/mutant cells were subjected to the Plk1 inhibitor (0–20 nm, 24 h), followed by an incubation period of 72 h in drug‐free medium. As shown in Fig. 4A,B, a dose‐dependent increase in β‐galactosidase staining was observed in all cell lines, accompanied by morphological readouts of cellular senescence. Strikingly, cells with functional p53 underwent significantly more senescence after treatment with volasertib compared to p53 nonfunctional cells (*P* < 0.021). A 9.77‐fold increase in β‐galactosidase staining was observed in A549 cells treated with 20 nm volasertib relative to untreated cells, while only a 2.47‐fold increase was detected in NCI‐H1975 cells (*P* < 0.001). Moreover, less flattened senescence‐like morphology was seen in A549‐920 and NCI‐H1975 cells compared to p53 wild‐type cells.

In order to confirm the effect of volasertib on cellular senescence, immunofluorescent staining for the DNA damage marker γ‐H2AX was performed. Although DNA damage could be observed in all cell lines after Plk1 inhibition, significantly more γ‐H2AX foci per cell were observed in p53 wild‐type cells compared to cells with nonfunctional p53 (*P* < 0.052) (Fig. 4C,D). For example, in A549 cells, an increase from 3.38 ± 019 foci per cell to 11.42 ± 2.28 foci per cell was observed after treatment with vehicle and 20 nm volasertib, respectively, while only a slight increase from 1.10 ± 0.14 foci per cell to 3.60 ± 0.33 foci per cell was detected in the NCI‐H1975 cell line. Therefore, our data indicate that cellular senescence induced by volasertib was mediated, at least partially, in a p53‐dependent way.

In order to further confirm the induction of cellular senescence after treatment with volasertib, western blot analysis using antibodies against p53 and p21 was performed (Fig. 4E). Consistent with the experiments described above, p53 levels were significantly upregulated in p53 wild‐type cell lines A549 (*P* = 0.039) and A549‐NTC (*P* = 0.036) after treatment with 20 nm volasertib, while this increase was not significant in p53 knockdown/mutant cells (*P* > 0.068). Similarly, an increase in p21 expression was observed after treatment with the Plk1 inhibitor; however, this increase was only significant in the A549‐NTC cell line (*P* = 0.053).

### 3.5 Transwell migration experiments revealed inhibition of cell migration by volasertib treatment

**Figure 5:**
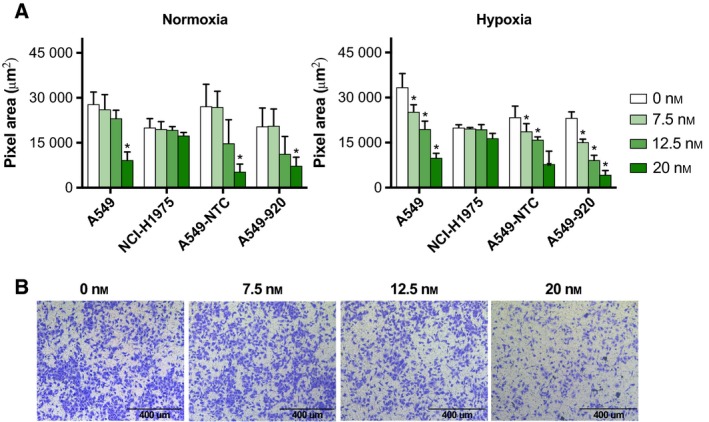
Volasertib has the potential to prevent migration of NSCLC cells. (A) Migratory behavior of the p53 wild‐type cell lines A549 and A549‐NTC, the p53 knockdown cell line A549‐920, and the p53 mutant cell line NCI‐1975 after treatment with volasertib (0–20 nm) for 24 h. Data are presented as mean pixel area from three independent triplicate experiments ± SD. *P‐value < 0.05 compared to untreated sample (one‐way ANOVA). (B) Pictures of migrated A549 cells after treatment with different concentrations (0–20 nm) of the Plk1 inhibitor (100×). Scale bars are shown on the pictures (400 μm).

Previous studies reported a correlation between Plk1 protein overexpression and lymphatic and distant metastases in several tumor types (Han *et al*., 2012; Kneisel *et al*., 2002; Zhang *et al*., 2013), suggesting that Plk1 might be involved in the metastatic process. Therefore, the effect of volasertib on the migration was investigated using a Transwell assay. As presented in Fig. 5A, downregulation of Plk1 using volasertib can block cell passing through the membrane in a dose‐dependent manner in three out of four tested cell lines. The pixel area occupied by A549 cells under normal oxygen tension was 27867 ± 4083 μm^2^ for untreated cells, while the highest volasertib concentration reduced the pixel area to 9145 ± 2764 μm^2^ (*P* < 0.001). Figure 5B demonstrates representative pictures of migrated A549 cells after treatment with 0 nm to 20 nm volasertib under normoxia. No significant differences in pixel area were observed when A549 cells were treated with lower volasertib concentrations (7.5–12.5 nm) under normal oxygen conditions (*P* > 0.458), whereas under hypoxia, a significant reduction in pixel area could be observed even after treatment with only 7.5 nm volasertib (*P* = 0.002). A stronger inhibitory effect of low volasertib concentrations was also seen when A549‐NTC and A549‐920 cells were exposed to oxygen deficiency (*P* < 0.040). Remarkably, volasertib had no effect on the migratory potential of *TP53* mutant NCI‐H1975 cells.

## 4 Discussion

Due to the important side effects of conventional microtubule‐targeting agents, more and more research focuses on regulatory proteins for the development of antimitotic agents. Plk1, an important player in cell cycle division, is considered as an important target in this research field. Volasertib, presently the most extensively studied Plk1 inhibitor, shows a promising cytotoxic effect in preclinical settings. However, only modest antitumor activity for volasertib was observed in clinical studies, with partial response in the minority of patients. Interestingly, an encouraging percentage of patients with solid tumors, varying from 26% to 44%, reached stable disease after treatment with volasertib (Van den Bossche *et al*., 2016). The discrepancy between the preclinical and clinical outcome prompted the research into the identification of molecules and mechanisms responsible for the sensitivity of Plk1 inhibitors. In this regard, p53 has previously been described as a potential predictive biomarker for Plk1 inhibition (Sanhaji *et al*., 2012, 2013). However, studies were often based on cell lines derived from various tumor origins or with a different cellular context, making comparisons difficult. In the present study, we examined the role of p53 as predictive biomarker for volasertib treatment in a panel of NSCLC cell lines differing in p53 status, including an isogenic knockdown cell line, under both normal and reduced oxygen conditions.

Treatment with volasertib significantly reduced cell proliferation in a dose‐dependent manner in all cell lines tested, with the highest IC_50_ values in p53 nonfunctional cells. Differences in sensitivity across the cell lines could not be attributed to different baseline Plk1 expression levels, as equal expression levels were detected in all cell lines. In literature, both the *in vitro* and *in vivo* growth inhibitory effect of volasertib has already been described in multiple human malignancies, including NSCLC (Brassesco *et al*., 2013; Krause *et al*., 2013; Pezuk *et al*., 2013; Rudolph *et al*., 2009). In accordance with our data, no correlations were found between proliferation rates, Plk1 expression levels, and sensitivity to Plk1 inhibition (Bogado *et al*., 2015; Gjertsen and Schoffski, 2015; Munch *et al*., 2015). This suggests that cell lines could have a different dependence on Plk1 or that genetic alterations, such as *TP53* mutations, could play an important role in the response to volasertib treatment. It has already been stated that the p53 and Plk1 pathway are highly intertwined in several ways (Louwen and Yuan, 2013). For example, it has been reported that p53 and its target genes p21, MDM2, and Bax were activated after Plk1 inhibition, suggesting that p53 plays a critical role in downstream signaling pathways (Tyagi *et al*., 2010). In line with our results, p53 wild‐type adrenocortical cells were more sensitive for Plk1 inhibition using siRNA or the small‐molecule inhibitor BI2536 compared to p53 mutant adrenocortical cells (Bussey *et al*., 2016). Besides, Sanhaji *et al*. (2012) examined the cytotoxic response of several Plk1 inhibitors, including volasertib, BI2536, and poloxin, in a series of isogenic cell lines differing in p53 status derived from breast cancer, lung cancer, colon cancer, and cervical carcinoma. They reported that p53‐deficient cells proliferated slower upon Plk1 inhibition compared to cells with functional p53 in all tumor types, including NSCLC. Nevertheless, this difference was not statistically significant. In addition, based on gene expression data of NSCLC cell lines treated with BI2536, volasertib, or GSK461364, Ferrarotto *et al*. (2016) did not find a statistically significant correlation between the *TP53* mutation status and the sensitivity to treatment with one of the three Plk1 inhibitors. Contrary, other research groups published that Plk1 inhibition using small interfering RNA (siRNA) or GSK461364 preferentially reduced the survival of p53^−/−^ cancer cells by inducing mitotic arrest, chromosome instability, and cell death, while p53 wild‐type cells activated a postmitotic checkpoint, leading to a pseudo G_1_ phase arrest and survival (Brassesco *et al*., 2013; Danovi *et al*., 2013; Degenhardt *et al*., 2010; McInnes and Wyatt, 2011; Yim and Erikson, 2014). It is essential to stress that these studies were based on cell lines derived from various tumor origins or with a different cellular context, which makes it difficult to draw conclusions about the impact of the p53 status on the growth inhibitory effect of Plk1 inhibition.

Remarkably, we observed a diminished sensitivity for volasertib when NSCLC cells were treated under hypoxic conditions (<0.1% O_2_). In literature, it has already been stated that hypoxic regions in solid tumors often contain viable cells that are intrinsically more resistant to treatment with radiotherapy or chemotherapy (Wouters *et al*., 2009). Nevertheless, to the best of our knowledge, no data are available on the *in vitro* effect of a Plk1 inhibitor under reduced oxygen tension. We hypothesize multiple mechanisms for the observed diminished cytotoxic effect. First, a significant increase in the percentage of G_1_ phase cells was noted after incubation in the hypoxic chamber. As Plk1 is a mitotic regulator, its expression and activity peak during the G_2_/M phase of the cell cycle, making it more difficult for volasertib to inhibit its target in G_1_ phase arrested cells. More recently, Ward *et al*. published that both Plk1 and Plk4 become hypermethylated upon oxidative stress [i.e., hypoxia and reactive oxygen species (ROS)] in a p53‐dependent manner, resulting in diminished protein levels and activity. Indeed, our results showed a decrease in baseline Plk1 protein expression when cells were cultured in the hypoxic chamber. As Plk1 protein levels are diminished under hypoxia, less Plk1 is available for binding with volasertib, resulting in less cytotoxicity. However, the influence of oxidative stress on the sensitivity for Plk1 inhibitors was not investigated (Liu, 2015). Secondly, HIF‐1α is reported to be a major player in the response to hypoxia, by acting as transcription factor for genes involved in cell proliferation, cell cycle, apoptosis, cellular metabolism, and angiogenesis, in order to promote cell survival. Of note, several research groups described a reciprocal negative relationship between HIF‐1α and the tumor suppressor p53. On the one hand, HIF‐1α has been reported to be upregulated under hypoxia, resulting in downregulation of p53 protein levels and stimulating cell survival. Otherwise, p53 has been shown to negatively regulate HIF‐1α protein levels, resulting in reduced transcriptional activity and induction of apoptosis (Hammond and Giaccia, 2006; Sermeus and Michiels, 2011). As such, HIF‐1α could be responsible for the reduced cytotoxic effect of volasertib under hypoxia. In the absence of functional p53, HIF‐1α can take the upper hand in the response to oxidative stress (Kamat *et al*., 2007). This is in line with our study results, which showed even more resistance to Plk1 inhibition in p53 knockdown (A549‐920) and mutant (NCI‐H1975) cells compared to p53 wild‐type (A549 and A549‐NTC) cells under hypoxia. Moreover, a strong trend for higher HIF‐1α expression levels was observed in p53 nonfunctional/mutant cells compared to p53 wild‐type cells when cells were treated with the Plk1 inhibitor under hypoxic conditions.

Finally, oxygen deficiency might activate survival pathways additional to Plk1 signaling (e.g., PI3K/AKT and/or MAPK/Erk), thereby further inhibiting programmed cell death.

Concerning the effect on cell cycle distribution, we have shown that treatment with volasertib provoked a clear mitotic arrest under both normoxia and hypoxia, as confirmed by an accumulation of cells in the G_2_/M phase of the cell cycle and immunofluorescent staining for pHH3. In literature, several studies linked Plk1 inhibition to an accumulation of cells with 4N DNA content, as seen by an increased G_2_/M peak in the DNA FACS profiles. Treatment with volasertib is known to result in the formation of monopolar spindles, leading to activation of the spindle assembly checkpoint (SAC) and prometaphase arrest (Bogado *et al*., 2015; Brassesco *et al*., 2013; Krause *et al*., 2013; Rudolph *et al*., 2009; Sparta *et al*., 2014). Interestingly, in our study, the observed mitotic arrest was more pronounced in p53‐defective cells compared to their p53 functional counterparts, at least under normoxia. This is in accordance with the study of Sanhaji *et al*., who demonstrated a stronger increase Plk1 and cyclin B expression in p53 wild‐type cells versus p53‐deficient cells after Plk1 inhibition (Sanhaji *et al*., 2012). Raab *et al*. demonstrated that prolonged activation of SAC, induced by high volasertib concentrations, resulted in downregulation of these mitotic proteins without SAC inactivation. The process of mitotic slippage was associated with repeated rounds of DNA synthesis without cell division, as indicated by an increase in the > 4N population (Raab *et al*., 2015). Nevertheless, in the present study, the occurrence of mitotic slippage is as good as negligible since only low concentrations of the Plk1 inhibitor were used and no increase in the > 4N population was observed after 24 h of treatment.

After 72 h of treatment, the cell cycle arrest resulted in apoptotic cell death under both normal and reduced oxygen conditions, as evidenced by (a) a sub‐G_1_ peak, (b) a higher percentage of Annexin V‐/PI‐positive cells, and (c) an increase in caspase 3/7 activity. Importantly, our results show that the p53 status might be an important factor in the balance between mitotic arrest and cell death induction. Although a dose‐dependent increase in apoptosis could be observed in all cell lines after treatment with the Plk1 inhibitor, the increase in cell death was significantly higher in A549 and A549‐NTC cells, which are both wild‐type p53. Similarly, others have reported that Plk1 depletion induced apoptosis in a p53‐dependent manner (Cholewa *et al*., 2017; Krause *et al*., 2013; Munch *et al*., 2015; Rudolph *et al*., 2009, 2015; Schwermer *et al*., 2017; Wissing *et al*., 2013). Furthermore, our results are in line with previous experiments of Sanhaji *et al*., who demonstrated that mitotic arrest (evidenced by increased expression levels of Plk1 and cyclin B) was noted in p53^−/−^ cells, while p53^+/+^ cells exhibited strong apoptosis (i.e., enhanced cleavage of poly(ADP)ribose polymerase (PARP) and cleaved caspase 3/7 activity) 48 h post‐treatment. As such, they also observed that cancer cells with wild‐type p53 induced more apoptosis upon Plk1 inhibition than cancer cells without functional p53 (Sanhaji *et al*., 2012). The underlying mechanisms for the induction of apoptotic cell death after Plk1 inhibition were further investigated in cholangiocarcinoma and lung cancer. In these studies, it was reported that expression of Mcl‐1, an antiapoptotic Bcl‐2 family member, was rapidly reduced in a post‐translational manner after treatment with Plk1 siRNA or volasertib (Fingas *et al*., 2013; Sekimoto *et al*., 2017). Furthermore, Sekimoto *et al*. demonstrated a decrease in the expression of KPNB1, a cell survival‐promoting protein, as one of the first steps during the induction of apoptosis after Plk1 inhibition in lung adenocarcinoma (Sekimoto *et al*., 2017). In addition, a correlation between Plk1 inhibition and mitotic catastrophe, another type of cell death involving the formation of micronuclei, was reported in NSCLC as a result of prolonged activation of the spindle checkpoint activation (SAC) upon treatment with BI2536 (Choi *et al*., 2015).

Notably, we are the first to describe premature cellular senescence as an important therapeutic outcome for volasertib treatment in NSCLC. Recently, a wide variety of anticancer treatments, including antimitotic agents, has been described to induce senescence‐like morphological and biochemical changes in tumor cells (Ewald *et al*., 2010; Roninson, 2003). The tumor suppressor p53 is designated as a major regulator of replicative senescence, but its role in treatment‐induced senescence (TIS) is less well‐defined. Our results indicated that induction of TIS after volasertib treatment is, at least partially, dependent on p53, as significantly more senescent cells were detected in the p53 wild‐type cell lines compared to p53‐deficient/mutant cells. This is consistent with the study of Kim *et al*. (2013), who reported that Plk1 knockdown using siRNA did not induce a senescent phenotype in p53 shRNA‐treated cells. Similarly, Driscoll *et al*. (2014) demonstrated more TIS in p53 wild‐type versus mutant colorectal and lung cancer cells after treatment with the Plk1 inhibitors MLN0905 or BI2536. From a therapeutic standpoint, it is noteworthy that cells that undergo cellular senescence are permanently growth arrested, but remain viable and metabolically active, thereby secreting multiple tumor‐promoting factors to adjacent tumor cells. As such, the significant percentage of NSCLC patients that reached stable disease after Plk1 inhibition most possibly resembles the induction of cellular senescence. In this view, the study from te Poele *et al*. (2002) linked TIS to stable disease rather than tumor regression in *in vivo* models of solid tumors.

Finally, there are also data available on the involvement of Plk1 in cancer cell migration and invasion. In previous studies, elevated Plk1 expression levels were correlated with invasion in several tumor types, such as colon carcinoma, bladder cancer, thyroid cancer, and lung cancer (Han *et al*., 2012; Li *et al*., 2017; Rizki *et al*., 2007; Wu *et al*., 2016; Zhang *et al*., 2013). Likewise, Wu *et al*. (2016) demonstrated that Plk1 overexpression in prostate epithelial cells triggered dramatic transcriptional reprogramming of the oncogenic cells via activation of the MEK1/2‐ERK1/2‐Fra1‐ZEB1/2 signaling pathway, leading to epithelial‐to‐mesenchymal transition (EMT) and stimulation of cell migration and invasion. In line with these results, a large‐scale integrated analysis of Plk1 inhibitor sensitivity showed that NSCLC cell lines with high epithelial–mesenchymal transition gene signature scores were more sensitive to Plk1 inhibitors than epithelial NSCLC cells. Moreover, induction of an epithelial phenotype by expression of the microRNA miR‐200 increased cellular resistance to volasertib (Ferrarotto *et al*., 2016). Another study in colorectal cancer showed that Plk1‐mediated invasion occurs via phosphorylation of vimentin (Ser82), which in turn regulates the cell surface levels of β‐integrin, a key player in cell adhesion (Rizki *et al*., 2007). Therefore, in the present study, we addressed the ability of volasertib to reduce migration of NSCLC cells. The migratory potential was significantly decreased in A549, A549‐NTC, and A549‐920 cells, but not in NCI‐H1975 cells. Inhibition of migration/invasion upon volasertib treatment was previously described by Brassesco *et al*. (2013) and Chakravarthi *et al*. (2016). Importantly, in these publications, the *TP53* status was not taken into account. As it has been demonstrated that both murine oviductal epithelial cells and endometrial cells harboring the *TP53* mutation (R273H) migrate easier compared to *TP53* wild‐type cells (Dong *et al*., 2007; Quartuccio *et al*., 2015), it is likely that the migratory activity of NCI‐H1975 cells was too abundant to be blocked by volasertib due to the gain‐of‐function mutation.

The identification of predictive biomarkers for the response to Plk1 inhibition is crucial to enhance antitumor activity in NSCLC patients. Most publications, including our study, are focused on the tumor suppressor p53. Nevertheless, previous studies also revealed influences of important oncogenes, such as KRAS, on the sensitivity to Plk1 inhibition. In these studies, cell lines with KRAS mutations were more sensitive to Plk1 inhibition than cell lines wild‐type for KRAS (Ferrarotto *et al*., 2016; Wang *et al*., 2016). In a clinical trial investigating the Plk1 inhibitor BI235, KRAS‐mutated patients showed numerically prolonged progression‐free survival, but statistical significance was not established (Breitenbuecher *et al*., 2017). As a result, the relationship between Plk1 and tumor suppressor/oncogenes is complicated and more research is warranted to explore their predictive roles.

## 5 Conclusion

In conclusion, our results show an important difference in the therapeutic effect of Plk1 inhibition in NSCLC cells with and without functional p53. Overall, the p53 wild‐type cell lines were more sensitive to volasertib treatment, suggesting that p53 could act as a potential predictive biomarker for Plk1 inhibition in NSCLC. Investigation of the underlying mechanisms revealed that while p53‐deficient/mutant cells were mostly arrested in the G_2_/M phase of the cell cycle, cell death and cellular senescence were predominantly induced in p53 wild‐type cells. Nevertheless, further research is warranted to elucidate the molecular pathways that are responsible for these differences in treatment outcome. As biomarker‐based patient selection could possibly improve the success rate of Plk1 inhibitors, it is warranted to include the *TP53* mutation status of patients in clinical trials testing volasertib treatment. More importantly, our results pave the way for new combination strategies with volasertib to further enhance antitumor efficacy. First, reactivation of mutant *TP53*, using small molecules (such as APR‐246 (PRIMA‐1^MET^)) that restore p53's wild‐type conformation, in combination with volasertib treatment could result in the induction of apoptotic cell death instead of a strong cell cycle arrest. Besides, it is crucial to further investigate the molecular pathways that are activated in senescent cells upon treatment with volasertib. New combination therapies with Plk1 inhibitors and agents eliminating senescent cells can possibly cause a shift from stable disease toward partial or clinical response in clinical trials investigating volasertib. As a result, there is still an intriguing window for improving patient outcome after Plk1 inhibition in patients with solid tumors, including NSCLC.

## Conflict of interest

J.B. Vermorken participated in advisory boards of Boehringer Ingelheim. The other authors declare that they have no conflict of interest.

## Author contributions

JVDB participated in the design of the study, carried out the *in vitro* experiments, analyzed data, performed statistical analysis, and drafted the manuscript. CD assisted the *in vitro* experiments, participated in the analysis and interpretation of the data, and contributed to draft the manuscript. IDP, VD, and JJ contributed to analysis and interpretation of the data and revised the manuscript. HL and CH assisted with the *in vitro* experiments and contributed to the analysis and interpretation of the data. PS, PP, JBV, and MP participated in the design of the study, supervised research, and revised the manuscript. AW and FL conceived the study, participated in the design and coordination of the experiments, and assisted to draft the manuscript. All authors read and approved the final manuscript.

## Supporting information


**Fig. S1.** P53 levels after treatment with cisplatin in order to confirm p53 shRNA transduction. P53 protein levels were determined using western blot after 72h treatment with 5 μM cisplatin, a known inducer of p53, in the A549 parental cell line, non‐template control cell line A549‐NTC, the p53 shRNA transduced cell line A549‐920 and the p53 mutant cell line NCI‐H1975. β‐Actin was used as an internal standard.
