# Supplementary figures and images for: In vitro study of the Polo‐like kinase 1 inhibitor volasertib in non‐small‐cell lung cancer reveals a role for the tumor suppressor p53
Source: Mol Oncol. 2019 Apr 5;13(5):1196–213. doi: 10.1002/1878-0261.12477 (PMC6487694; doi:10.1002/1878-0261.12477)

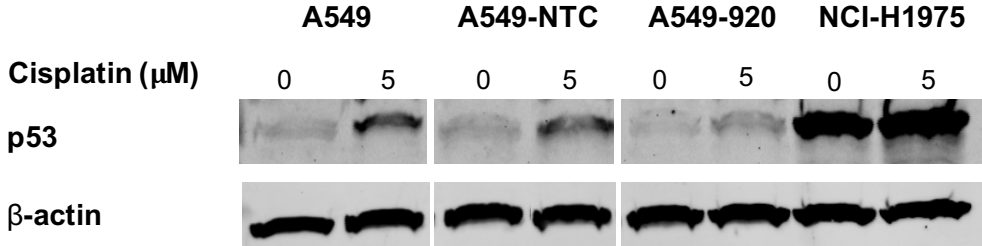

Supplement: Supplementary file 1 — Fig. S1. P53 levels after treatment with cisplatin in order to confirm p53 shRNA transduction. P53 protein levels were determined using western blot after 72h treatment with 5 μM cisplatin, a known inducer of p53, in the A549 parental cell line, non‐template control cell line A549‐NTC, the p53 shRNA transduced cell line A549‐920 and the p53 mutant cell line NCI‐H1975. β‐Actin was used as an internal standard. [file MOL2-13-1196-s001.pdf]
